# H.R.H. the Prince of Wales

**Published:** 1915-06-26

**Authors:** 


### The Hospital

The Workers' Newspaper of Administrative Medicine and Institutional Life, Administration, National Insurance and Health.

No. 1515, Vol. LVIII. SATURDAY, JUNE 26, 1915. PRICE ONE PENNY

### H.R.H. THE PRINCE OF WALES.

Wednesday, June 23, 1915, should prove memorable and important to British charities, and especially to the voluntary hospitals, for on that day the Prince of Wales attained his majority without any official ceremony or public demonstration, owing to the war. The personality of the Prince is known to the British people, and particularly to Londoners. Wherever the Prince has gone, and amongst all classes with whom he has mingled, both at home and abroad, he has won golden opinions and confidence. We believe that his education from boyhood upwards has been conceived upon a plan far wiser and better calculated to bring out all that is best in his character than probably any prince the world has ever known. When thirteen he went to Osborne, two years later to Dartmouth, and subsequently was appointed as a midshipman to the *Hindustan*. Three years later he went to Paris as the Earl of Chester, studying French and the institutions and people of France. He was everywhere received, like his grandfather, with cordiality and interest. On his return he became a student at Magdalen College, Oxford, where he lived the life of an ordinary undergraduate in every particular. One great feature of his character is his keenness, and this was shown by the progress he made at Oxford and by the energetic way in which he threw himself into games and sports. He showed an equal keenness in the Navy, and in everything he has been eager to learn, to gain knowledge, and the power to accomplish. The more active the work, the more difficult the task, the more he appreciates it. Keen on the Navy, when the war broke out he became anxious to see active service. On August 8 he was gazetted a second lieutenant of the Grenadier Guards, and immediately afterwards he entered with zest upon the requisite training to fit him for active service. Three months later he was promoted to lieutenant, and gazetted to Sir John French's Staff at the seat of war. His record there can be gathered from the following description taken from a soldier's letter:—"Our Prince is a soldier and a man, with a bigger heart than a lot who are hanging back in Great Britain."

It will be seen that the Prince has lived a strenuous, devoted life throughout his youth, and that he enters upon manhood with a record which has won for him everywhere the affection and confidence of all who had the pleasure of being associated with him at school, at college, in the Navy and the Army, and throughout his whole career. The *Times*, in an appreciative notice, declares: "The public loves its Prince for just so much as there has been in him of the normal, shy, and natural boy, as well as for the sense of Royalty which has exalted him above the common when need was." The Prince spent his twenty-first birthday at the seat of war. There were no public rejoicings or outward evidence of popular enthusiasm on His Royal Highness's coming of age. But never was a royal Prince's birthday so well kept in the homes of the people with every manifestation of genuine affection and loyalty.

Queen Victoria, King Edward, and King George, with the members of the Royal Family, have always been strenuous upholders of the voluntary system for hospitals. Personal service in the cause of the sick has been a marked feature in the lives of King Edward and King George, and we have no doubt that when the time arrives for the Prince of Wales to take up his permanent responsibilities after the war, he will follow the examples of his grandfather and father with keenness and conviction. Their labours have provided for him in the field of charity a goodly heritage. In the ordinary course under the Act he will become President of King Edward's Hospital Fund for London; and, under the Charter, Grand President of the League of Mercy. These two important organisations have proved of vast importance to the voluntary hospitals of the Metropolis and the Home Counties, and so long as they are maintained under efficient management they cannot fail to be of great assistance to some of our greatest voluntary institutions. When the Hospital Sunday Fund proved inadequate as a means to raise sufficient money to make good the deficiency between income and expenditure of the London hospitals, the King's Fund was established, and has nobly accomplished this object. It has had a quickening influence for good in the administration and management of all institutions to which grants have been made.

Nowhere will the Prince receive a warmer welcome than in the field of charity, and nowhere, we are confident, will his services and personal influence be more beneficial or grateful.

